# Efficacy of 104-week Telbivudine-based optimization strategy in patients with HBeAg-negative chronic hepatitis B virus infections

**DOI:** 10.1186/s12879-020-05642-y

**Published:** 2020-12-07

**Authors:** Weiqiang Gan, Jianguo Li, Chunlan Zhang, Xuefu Chen, Chaoshuang Lin, Zhiliang Gao

**Affiliations:** 1 Department of Infectious Disease, The Third Affiliated Hospital of Sun Yat-sen University, 600 Tianhe Road, Tianhe District, Guangzhou, 510630 Guangdong Province China; 2 First Department of Liver Disease, Guangzhou Eighth People’s Hospital, Guangzhou, 510000 Guangdong Province China; 3 Department of Infectious Disease, Guangdong General Hospital, Guangzhou, 510000 Guangdong Province China

**Keywords:** Chronic hepatitis B, eGFR, HBeAg, Telbivudine

## Abstract

**Background:**

Evaluate the safety and efficacy of 104-week regimen of Telbivudine(LdT)-based optimization strategy for Chinese patients who have chronic hepatits B(CHB) with HBeAg-negative.

**Methods:**

This multi-center, open-label, prospective study enrolled 108 HBeAg-negative CHB patients who received LdT (600 mg/day) for 24 weeks, Adefovir (ADV) was added if HBV DNA remained detectable at week 24, otherwise LdT was maintained to use until 104 weeks. HBV DNA, alanine amino transferase (ALT), hepatitis B surface antigen(HBsAg), creatinine kinase(CK), and estimated glomerular filtration rate (eGFR) were measured, safety was assessed.

**Results:**

Eighty-eight patients (81%) had HBV-DNA undetectable at 24 weeks and maintained to receive LdT monotherapy until 104 weeks, whereas the other 20 patients had HBV-DNA detectable and ADV was used in combination. For all patients, 72% of patients reached ALT normalization at 24 weeks, which increased to 80% at 52 weeks and 104 weeks, respectively.. 81% of total patients had undetectable HBV-DNA at 24 weeks, 92% at 52 weeks, and 94% at 104 weeks. The HBsAg titre declined steadily from baseline to 104 weeks (3.62 vs. 2.98 log10 IU/mL, *p* < 0.05), and the eGFR increased steadily from baseline to 104 weeks (92.9 vs. 104.4 mL/min/1.73 m^2^, *p* < 0.05). Although 79 patients (73%) had at least one time of elevated CK, most of these patients had CK elevated in Grade 1/2.

**Conclusions:**

LdT was well tolerated and effective, and 94% of patients achieved virological suppression after 104 weeks.

**Trial registration:**

This study was registered in clinicaltrials.gov on January 31, 2012 and the ID No. was NCT01521975.

## Background

Chronic hepatitis B virus (HBV) infection is a major public health problem worldwide. An estimated 240 million people have chronic HBV infections [1], and about 75% of them reside in the Asia-Pacific region [2]. HBV infection causes more than one million deaths every year due to acute or chronic outcomes, including end-stage liver disease, cirrhosis, and hepatocellular carcinoma (HCC) [3, 4]. Therefore, more aggressive antiviral therapies are needed to manage and control it.

In recent decades, regulatory agencies have approved several antiviral agents for HBV infections treatment, including interferon and several oral drugs (such as telbivudine [LdT], lamivudine [LAM], tenofovir [TDF], entecavir, and adefovir [ADV]) [5–7]. Among the oral antiviral agents, several major guidelines for chronic hepatitis B recommended entecavir and tenofovir as first-line therapy for CHB [3, 8, 9]. However, tenofovir is not available but entecavir is not affordable in some low-income countries, which limited the first-line therapy options for many patients. In recent years, with the listing of tenofovir, the price of entecavir in China has dropped significantly.

LdT is a β-L-nucleoside analog which is chose to treat patients with chronic HBV infections who have active viral replication and abnormal levels of ALT. LdT has better efficacy than lamivudine and has a higher HBeAg seroconversion rate in HBeAg-postive HBV patients compared with other oral antiviral agents [10]. ,However, there has few researches regarding the efficacy of LdT in HBV patients with HBeAg-negative. This study evaluated the safety and efficacy of LdT -Based Optimization Strategy in patients with HBV who were HBeAg-negative.

## Methods

### Study design

This was a prospective, 104-week, multicenter, single-arm, open-label, phase IV, long-term trial that enrolled 154 patients from April 2012 to July 2014 among three hospitals in Guangdong Province, China (The Third Affiliated Hospital of Sun-Yet-Sen University, Guangdong General Hospital, and The Eighth People’s Hospital of Guangzhou). All enrolled patients received sequential therapy with supportive care during the whole study. Each patient received single oral tablet of LdT (600 mg) daily for the first 24 weeks. ADV was added if HBV DNA remained detectable at week 24, otherwise LdT was maintained to use until 104 weeks. Patients received LDT monotherapy with virological breakthrough were added with adefovir, otherwise TDF was chose as rescue treatment. Clinical, laboratory, and adverse event assessments were collected every 12 or 16 weeks from baseline (week 0) to week 104. This study conformed to the ethics principles of the Declaration of Helsinki and Good Clinical Practice and according to the regulatory requirements of China. It also obtained ethical approval from the Institutional Review Board (IRB) of the Third Affiliated Hospital of Sun-Yat-Sen University, IRB of Guangdong General Hospital and IRB of the Eighth People’s Hospital of Guangzhou. Written informed consent have been obtained from all participants. This study was registered in clinicaltrials.gov on January 31, 2012 and the ID No. was NCT01521975.

### Inclusion and exclusion criteria

Inclusion criteria: (1) patients had HBeAg-negative CHB; (2) patients aged 18–65 years-old; (3) patients had detectable HBV S-antigen (HBsAg) at screening visit, with serum HBV-DNA level above 4 log_10_ IU/mL; (4) patients had ALT level at 2 to 10-fold above the upper limit than normal (ULN) of 35 U/L; (5) patients had ALT level at less than 10-fold ULN if they were complicated with liver cirrhosis. Exclusion criteria: (1) patients had coinfection with hepatitis C virus, hepatitis D virus, or HIV, decompensated liver cirrhosis or HCC; (2) patients received nucleoside analogs, immunosuppressive drugs, or other antiviral drugs; (3) patients had history of tumor.

### Index detection

HBV m quantitative time resolved fluorescence diagnostic kit was provided by Suzhou Xinbo Biotechnology Co., Ltd. (Suzhou, China) and analyzed by 1235 automatic time-resolved fluoroimmunoassay system (American PE company). HBV DNA quantitative detection kit was provided by Sun Yat sen University Da’an gene Co., Ltd. (Guangzhou, China) and analyzed by Gene-Amp5700 fluorescent PCR detector (American PE company). Serum CK, creatine kinase, ULN, ALT and eGFR were detected by automatic biochemical analyzer with commercial kits (Nanjing Jiangcheng Bioengineering Institute, Nanjing, China).

### Efficacy assessment

The primary endpoint was undetectable HBV-DNA (< 20 IU/mL) at week 52 and 104. The secondary endpoint was ALT normalization at week 24, 52, and 104 and loss of HBsAg seroconversion at week 52 and 104. Virological breakthrough was also recorded.

### Safety assessment

The safety assessment consisted of adverse events (AEs) monitoring from clinical and laboratory examinations and their relationships with this study, based on the investigators’ assessment. The safety population included all patients who received at least one dose of the study medication (intention to treat [ITT] population) and had at least one post-baseline safety assessment.

### Statistical analysis

All statistical analyses were performed using SPSS (SPSS Statistics Version 19, IBM). Continuous variables were described as means and standard deviations (SD) and categorical variables (e.g. age and gender) as number and percentages. Significant differences between groups were evaluated using one-way analysis of variance. Differences between groups were considered statistically significant at *P* < 0.05.

## Results

### Patient enrollment and baseline characteristics

The trial initially enrolled 154 patients, 66 patients still had detectable HBV DNA at week 24, who were introduced the Roadmap strategy to all of the patients and suggested to add ADV, 25 patients rejected the suggestion for higher cost, the other 21 patients declined the strategy considering more side effects, these 46 patients were excluded from the study. The other 20 patients received ADV add-on therapy, totally 108 patients were eligible for efficacy analysis finally. The mean baseline level of HBV-DNA was 6.0 log_10_ IU/mL and the mean level of ALT was 135 U/L (Table 1).

**Table 1:** Baseline demographic and disease characteristics of CHB patients treated with telbivudine (*n* = 108)

Characteristic	N (SD or %)
Age, years	40 (±10)
Sex, male, n (%)	87 (81%)
HBV DNA, log_10_IU/mL	6.0 (±1.3)
HBsAg titre, log_10_IU/mL	3.6 (±0.5)
ALT, U/L	135 (±131)
CK, U/L	129 (±61)

### Efficacy: biochemical response

**Fig. 1:**
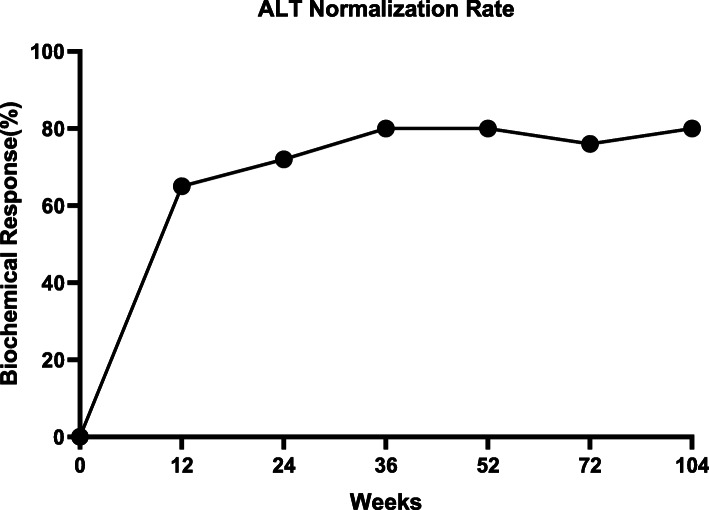
ALT Normalization Rate

After 12 weeks of LdT therapy, 70 patients (65%) had ALT levels below the ULN (Fig. 1), and the ALT declined by an average (±SD) of 102 ± 134 U/L relative to baseline (*p* < 0.05). The number of patients with ALT levels below the ULN continued to increase throughout the study period (week 24: *n* = 78, 72%; week 52: *n* = 86, 80%; week 104: *n* = 86, 80%).

### Efficacy: virological response

**Fig. 2:**
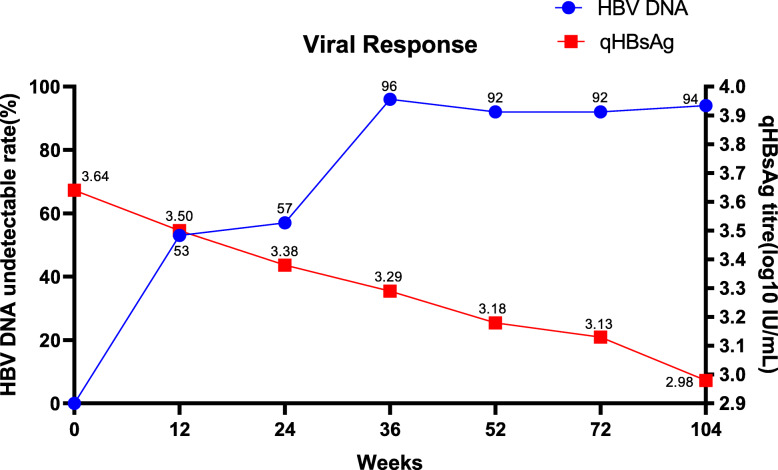
Viral Response of HBV DNA and qHBsAg

At week 12, 81out of 154 (53%) patients had undetectable HBV-DNA (< 20 IU/mL), and the HBV-DNA declined an average of 5.1 log_10_ IU/mL (range: 4.8 to 5.5 log_10_ IU/mL, *p* < 0.05) (Fig. 2). At week 24, 88 out of 154 (57%) patients had undetectable HBV-DNA. Sixty-six patients had detectable HBV-DNA and ADV was added to 20 patients, the other 46 patients were excluded from the Roadmap study and the subsequent treatment and observation. The virological response using LdT-based optimization strategy was high throughout the study period (week 36: 96%, 104/108; week 52: 92%, 99/108; week 102: 94%).

### Efficacy: serologic response

There was a significant decrease in the mean HBsAg titre from baseline until week 24 (3.62 vs. 3.38 log_10_ IU/mL; *p* < 0.001) (Fig. 2). This decline tendency continued to week 52 (3.18 log_10_ IU/mL, *p* < 0.001) and week 104 (2.98 log_10_ IU/mL, *p* < 0.001). Only one patient experienced HBsAg loss without HBsAg seroconversion.

### Virological breakthrough

A total of 6 patients (5.6%) had virological breakthroughs during the 104-week therapy period (Table 2). Among these 6 patients, 1 had virological breakthrough at week 36, 5 had breakthroughs at week 52. M204V mutation was found in 3 patients, L180M mutation in 1 patient and M204I mutation in 2 patients. We switched 3 of these 6 patients to TDF, then these 3 patients had undetectable HBV-DNA after 24 weeks. ADV was added to the other 3 patients, 2 of them had undetectable HBV DNA after 24 weeks, and 1 patient had undetectable after 48 weeks.

**Table 2:** Virological breakthrough during the study

Patient (P^n^)	Group	Breakthrough time (Week)	Mutation	Rescue therapy
P^1^	LDT	36	M204I	LDT + ADV
P^2^	LDT	52	M204I	LDT + ADV
P^3^	LDT	52	M204V	LDT + ADV
P^4^	LDT	52	L180M	LDT + ADV
P^5^	LDT + ADV	52	M204V	TDF
P^6^	LDT + ADV	52	M204V	TDF

### Changes in eGFR

**Fig. 3:**
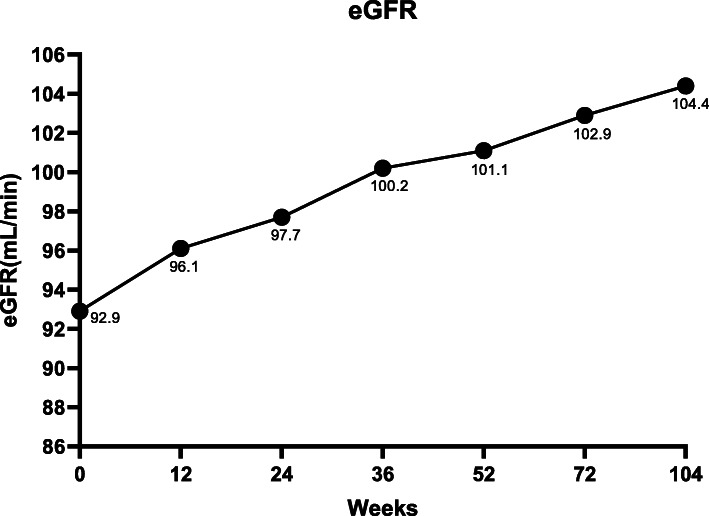
eGFR levels

The baseline eGFR was 92.9 mL/min/1.73 m^2^, and this increased to 97.7 mL/min/1.73 m^2^ at week 24 (*p* < 0.001), then up to 104.4 mL/min/1.73 m^2^ at week 104 (*p* < 0.001) (Fig. 3).

### Safety evaluation

A total of 45 (29%) patients reported AEs who were related to the study medication, but with no deaths. Overall, there were 6 serious AEs (3.9%): pneumonia for 3 patients, urinary tract infections for 2 patients, and gastric ulcer for 1 patient. All of these serious AEs were resolved with supplemental treatments, and cessation of LdT therapy was not necessary, so we considered that none of them was related to the study medication.

An increasing number of patients had elevated serum CK during the study period (Table 3). A total of 79 (51%) patients had CK elevation in Grade 1 at least during the study period, but most of them only had elevations in Grade 1/2. Although 10 of 108 patients (9%) had CK elevation in Grade 3/4, all of them had these elevations during only one visit. The patients who had CK elevation in Grade 4 at week 52 exercised the day before blood was drawn, and he returned to CK elevation in Grade 2 after 4 weeks. Among all 79 patients with elevated CK levels, 17 patients were complicated with myalgia that was resolved gradually.

**Table 3:** CK elevation during telbivudine treatment

CK	Baseline	Week 12	Week 24	Week 36	Week 52	Week 72	Week 104
Grade 1, n	14	22	26	29	38	31	42
Grade 2, n	0	3	3	14	19	13	16
Grade 3, n	0	0	0	1	2	6	0
Grade 4, n	0	0	0	0	1	0	0
Total, n	14	25	29	44	60	50	58

## Discussion

Many major guidelines for liver diseases, including the American Association for the Study of Liver Diseases (AASLD) and the European Association for Study of the Liver (EASL), only recommend entecavir or tenofovir as first-line therapy for treatment patients with HBV [3]. However, many patients from the Asia-Pacific region do not have access to these drugs forced by money. Thus, lamivudine, LdT, and adefovir are still widely used in treatment of HBV.

The GLOBE study indicated that LdT had better efficacy than lamivudine in terms of virological response, serologic response, and drug resistance in CHB patients with HBeAg-positive [11, 12]. After treatment for 2 years in GLOBE study, 82% of patients had non-detectable viremia. After 104 weeks of LdT-based optimizedd strategy, our population had a higher response rate (94%), although the research illustrated that response to entacavir was even greater (96.5%) [13]. As a potent antiviral agent, LdT has proven efficacy in suppressing HBV replication, and has advantage in HBeAg seroconversion over other antiviral agents in HBeAg-positive patients with CHB [14]. However, few studies have examined the effect of LdT in HBeAg-negative patients with CHB. Our results showed that LdT-based optimization strategy was effective, and 80% of patients turned to normal ALT levels and 94% patients became to undetectable HBV-DNA at week-104.

A major concern regarding LdT therapy had high rate of resistance. Liaw, et al. reported the overall resistance rate was 25.1% after 2 years of LdT treatment for CHB patients [12], and the resistance rate was 10.8% for HBeAg-negative patients. Combination therapy might reduce drug resistance. Liu, et al. reported the incidence of drug resistance was 4% by using LdT plus ADV at week 48, 5.3% at week 96, 10% at week 144, and 11.6% at week 192 for HBeAg-positive patients with CHB [15]. In order to decrease the resistance rate, we utilized a Roadmap strategy. The 2-year drug resistance rate was 5.6% in our trial, much lower than the related research conclusions. We found that the accumulative incidence of drug resistance might increase with treatment duration, so more long-term data are needed in the future.

Another concern biomarker is CK elevation during LdT therapy, which have been reported in many previous studies [16, 17]. Chen, et al. studied 527 CHB patients and reported that 60.91% of them had CK elevation [18], which was similar with our result (55%). Few studies have reported the management and prognosis of CHB patients who had CK elevation in Grade 3/4 during LdT treatment, especially those who continued with this therapy. Our results reassuringly showed that when such patients continue LdT therapy, their CK levels would gradually normalize. Very few of our patients had musculoskeletal symptoms. These results implied that patients with CK elevation in Grade 3/4 might safely in continuous LdT therapy under monitoring closely.

HBsAg loss or seroconversion often leads to cessation of nucleoside treatment [3], and there contains special relationship of HBV genotypes and the rate of spontaneous or antiviral therapy-associated HBeAg seroconversion. For example, genotype C patients endure delayed HBeAg seroconversion and thus have a longer duration of high viral load than genotype B patients. Therefore, genotype C patients are correspondingly more prone to develop advanced fibrosis, cirrhosis and HCC than genotype B patients. And the presence of HBV mutants, such as precore and basal core promoter mutations, may affect the response to antiviral therapy in HBeAg-positive disease. Many studies demonstrated that HBsAg decreasing was related to better antiviral therapy outcome [19]. Our study showed that LdT-based optimization strategy could decrease the HBsAg level though no patients had loss of HBsAg. The duration of treatment in our study was only 2 years, so continued monitoring of the dynamics of HBsAg was necessary for longer treatment. Meanwhile, several biomarkers predicting HBeAg seroconversion have been identified. Such as the baseline total anti-HBc levels could predict HBeAg seroconversion in HBeAg-positive CHB patients receiving antiviral therapy [20, 21]. In addition, host genetic polymorphisms have been reported to predict response to pegylated interferon in HBeAg-positive patients [22]. And recent clinical trials revealed that patients receiving pegylated interferon combined or add-on nucleos(t)ide analogue had significantly higher HBeAg seroconversion rates than those receiving nucleos(t)ide analogue monotherapy [23, 24].

The quantitative detection of HBV serum markers and HBV DNA plays a complementary role in the diagnosis of HBV infection, which makes the diagnosis more accurate. It has been found that il-12a rs568408 is related to the seroconversion of Entecavir in the treatment of HBeAg. Recent studies have shown that IL-21 could promote the activation of various immune cell functions in the process of HBV infection. IL-21 gene polymorphism was related to HBV susceptibility. In addition, IL-21 expression was closely related to HBV genotype, HBV clearance, HBeAg seroconversion, HBV related cirrhosis, liver failure, liver cancer and autoimmune dieases. CHB can lead to chronic kidney disease, because of the accumulation of immune compounds in kidney. Gane, et al. found that LdT might increase eGFR by 8.5% for 4–6 years, and this effect was unrelated to its antiviral action [25], implying a direct influence of LdT to renal function. Our study also showed that LdT therapy improved eGFR. Thus, LdT might become a good treatment option for CHB patients, especially those with high risk of developing chronic kidney disease (i.e. those with high blood pressure or diabetes),.

## Conclusion

In conclusion, the current study showed that 104 week LdT-based optimization strategy was well-tolerated and effective in HBeAg-negative CHB patients, and that most patients achieved virological suppression after 104 weeks.

## Data Availability

The datasets used during the present study are available from the corresponding author upon reasonable request.

## Abbreviations

CHB: Chronic hepatits B
ADV: Adefovir
ALT: Alanine amino transferase
HBsAg: Hepatitis B surface antigen
CK: Creatinine kinase
eGFR: Estimated glomerular filtration rate
HBV: Chronic hepatitis B virus
HCC: Hepatocellular carcinoma
LdT: Telbivudine
LAM: Lamivudine
TDF: Tenofovir
ETV: Entecavir
HBsAg: HBV S-antigen
ULN: Upper limit than normal
AEs: Adverse events
ITT: Intention to treat
AASLD: The American Association for the Study of Liver Diseases
